# Analysis of *C. difficile* infection–related outcomes in European participants in the bezlotoxumab MODIFY I and II trials

**DOI:** 10.1007/s10096-020-03935-3

**Published:** 2020-06-06

**Authors:** Emilio Bouza, Oliver A. Cornely, Antonio Ramos-Martinez, Robert Plesniak, Misoo C. Ellison, Mary E. Hanson, Mary Beth Dorr

**Affiliations:** 1 University Complutense of Madrid, Madrid, Spain; 2 Faculty of Medicine and University Hospital Cologne, Department I of Internal Medicine, Excellence Center for Medical Mycology (ECMM); Cologne Excellence Cluster on Cellular Stress Responses in Aging-Associated Diseases (CECAD); Clinical Trials Centre Cologne (ZKS Köln), University of Cologne, Cologne, Germany; 3 Hospital Universitario Puerta de Hierro-Majadahonda, Madrid, Spain; 4 University of Rzeszów Medical Center, Łańcut, Poland; 5 Merck & Co., Inc., 2000 Galloping Hill Road, Kenilworth, NJ 07033 USA

**Keywords:** Bezlotoxumab, *Clostridioides difficile* infection, CDI recurrence, Rehospitalization

## Abstract

The MODIFY I/II trials demonstrated that bezlotoxumab, a human monoclonal antibody against *Clostridioides difficile* toxin B, given during antibiotic treatment for *Clostridioides difficile* infection (CDI) significantly reduced *C. difficile* recurrence (rCDI) in adults at high risk for rCDI. Efficacy of CDI-directed intervention may depend on ribotype regional epidemiology, and patient characteristics. This post hoc analysis assessed the efficacy of bezlotoxumab in the subgroup of MODIFY I/II trial participants enrolled in Europe. Data from the bezlotoxumab (10 mg/kg single intravenous infusion) and placebo (0.9% saline) groups from MODIFY I/II were compared to assess initial clinical cure (ICC), rCDI, all-cause, and CDI-associated rehospitalizations within 30 days of discharge, and mortality through 12 weeks post-infusion. Of 1554 worldwide participants, 606 were from Europe (bezlotoxumab *n* = 313, 51%; placebo *n* = 292; 48%). Baseline characteristics were generally similar across groups, although there were more immunocompromised participants in the bezlotoxumab group (27.2%) compared with placebo (20.1%). Fifty-five percent of participants were female, and 86% were hospitalized at randomization. The rate of ICC was similar between treatment groups. The rate of rCDI in the bezlotoxumab group was lower compared with placebo among European participants overall, and among those with ≥ 1 risk factor for rCDI. Bezlotoxumab reduced 30-day CDI-associated rehospitalizations compared with placebo. These results are consistent with overall results from the MODIFY trials and demonstrate that bezlotoxumab reduces rCDI and CDI-associated rehospitalizations in European patients with CDI. MODIFY I/II (NCT01241552 and NCT01513239)

## Introduction

*Clostridioides difficile* infection (CDI) is one of the most frequently reported hospital-acquired infections (HAIs) in European countries, accounting for 3.6% of all HAIs and 48% of all gastro-intestinal infections, both HA and non-HA (excluding hepatitis) [1]. With such high incidence rates, healthcare resource use and attributable financial burden of CDI are significant, mainly driven by length of hospital stay [2–4]. Retrospective cost analyses in various European countries estimated mean CDI attributable direct costs per hospital stay of €4396 to €14,023 [5–7]. The majority of primary CDI cases respond to antibiotic treatment; however, recurrence (rCDI) occurs in 25% of these cases with a 38% to 45% chance of subsequent recurrences [5, 8–12].

The phase 3 MODIFY I and II trials demonstrated that bezlotoxumab, a human monoclonal antibody against *C. difficile* toxin B, when given during standard of care (SOC) antibiotic treatment for an active CDI, significantly reduced rCDI in adults at high risk for rCDI [13, 14]. Healthcare practices and reimbursement methods differed across the countries that enrolled patients in the MODIFY trials. For example, European participants were more frequently treated in an inpatient setting compared with participants in North America. Additionally, the distribution of strains of *C. difficile* isolated from European participants differs from strains identified in North American participants, with a lower percentage of European participants infected with the 027 strain [15–18]. Given these differences, the objective of this analysis was to assess the efficacy of bezlotoxumab in preventing rCDI, as well as other secondary efficacy outcomes, in the subgroup of participants enrolled in the European region.

## Materials and methods

**Fig. 1:**
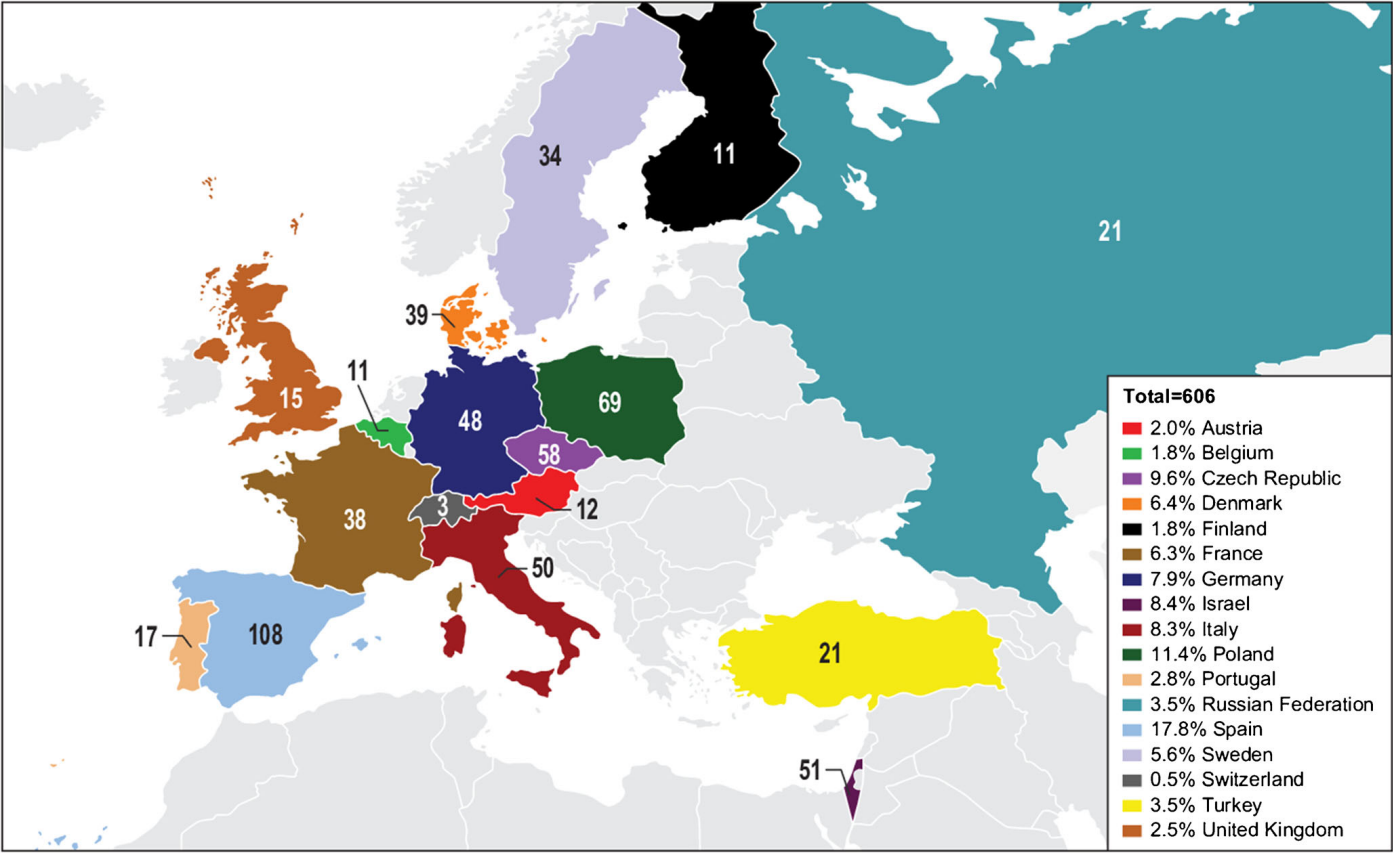
Number and proportion of participants enrolled in the European region shown by country in MODIFY I and MODIFY II (mITT population; numbers represent n’s)

The MODIFY I/II (NCT01241552 and NCT01513239) trials were randomized, double-blind, placebo-controlled, multicenter, phase 3 studies conducted from November 2011 through May 2015 at 322 sites in 30 countries, including 17 countries in the European region (Fig. 1). Both MODIFY I and II were conducted in accordance with the Good Clinical Practice guidelines and the Declaration of Helsinki. The protocols and amendments were approved by the institutional review board or independent ethics committee at each study site. Written informed consent was provided by all participants before the trial began.

Adult participants with primary or rCDI were receiving antibiotic treatment for CDI (metronidazole, vancomycin, or fidaxomicin, selected by the treating physician) for 10–14 days. Eligibility criteria were described previously [14]. CDI was defined as diarrhea (≥ 3 unformed bowel movement [types 5 to 7 on the Bristol stool scale] [19] in 24 h) associated with a positive local laboratory stool test result for toxigenic *C. difficile*. Eligible participants received a single 60-min infusion of bezlotoxumab 10 mg/kg or a placebo (0.9% saline) while continuing to receive antibiotic treatment. Randomization was stratified by oral antibacterial treatment (metronidazole, vancomycin, or fidaxomicin) and hospitalization status (inpatient or outpatient). Participants recorded their daily loose stool counts for 12 weeks and provided a stool sample for testing if they had a return of diarrhea.

## Population, endpoints, and statistical analysis

The analysis included the subpopulation of MODIFY I/II participants enrolled by investigators residing in the European region. The modified intent-to-treat (mITT) population was the analysis population for the initial clinical cure (ICC) and sustained clinical cure (SCC) endpoints and included all randomly assigned participants who received study infusion, had a positive baseline stool test for toxigenic *C. difficile*, and initiated standard of care (SOC) antibacterial therapy prior to or within 1 day after receiving study treatment. The population was further categorized based on known risk factors for rCDI or CDI-related adverse outcomes that were prespecified in the protocols: age ≥ 65 years, history of CDI in the previous 6 months, compromised immunity, severe CDI (Zar score ≥ 2 points) at the time of randomization, and isolation of a strain associated with poor outcomes (ribotypes 027, 078, or 244). ICC was defined as SOC given for ≤ 16 days and no diarrhea on the two consecutive days after SOC end. The primary endpoint was rCDI, defined as new diarrhea associated with toxigenic *C. difficile* in stool within 12 weeks following infusion in participants who had achieved ICC (clinical cure population). SCC was defined as ICC and no rCDI during the 12-week follow-up period. The rates of all-cause and CDI-related rehospitalization within 30 days of antibiotic treatment were assessed in mITT participants who were inpatients at the time of randomization, were subsequently discharged, and then were rehospitalized within 30 days of discharge. Rehospitalizations were characterized as CDI-associated if they were due to rCDI or if rCDI occurred during the rehospitalization. The all patients as treated (APaT) population consisted of all randomized patients who received an infusion of study medication and was used for the analysis of mortality. Participant disposition and baseline demographic and clinical characteristics of the mITT population were summarized descriptively using frequencies and percent for each treatment group unless specified otherwise. Observed rCDI rates among participants who achieved ICC, ICC rates, and SCC rates along with rate differences between treatment groups and their 95% confidence intervals (CI) are presented. The 95% CIs are based on Miettinen and Nurminen’s method [20]. The nonparametric Kaplan-Meier (KM) method was used to estimate the distribution of time to CDI recurrence for each treatment group. Other outcomes of interest during the 12-week follow-up period are summarized descriptively using frequencies and percent for each treatment group.

## Results

There were 1554 participants in the MODIFY trials; 606 were enrolled at a European site, including 313 in the bezlotoxumab group (51.7%) and 293 in the placebo group (48.3%). Figure 1 shows the distribution of participants by European country. Baseline characteristics were generally similar across groups (Table 1), although compared with placebo, there were more immunocompromised participants (27.2% vs 20.1%) and participants with 2 prespecified risk factors for rCDI (43.5% vs 33.9%) in the bezlotoxumab group. Conversely, there were approximately 6% more participants with renal impairment, 1 prespecified risk factor for rCDI, and a hypervirulent strain (i.e., ribotypes 027, 078, or 244) in the placebo group compared with the bezlotoxumab group. Slightly more than half (54.8%) of the participants were female, and 86.0% of all enrolled patients were hospitalized at the time of randomization.

**Table 1:** Baseline demographics and clinical characteristics (mITT population)

	Bezlotoxumab (*n* = 313)	Placebo (*n* = 293)
Demographics		
Mean age (years (SD))	65.7 (17.0)	66.9 (16.8)
Median	68	70
Range	19-97	18-96
18 to < 50 years	47 (15.0)	45 (15.4)
50 to < 65 years	82 (26.2)	63 (21.5)
65 to < 80 years	113 (36.1)	109 (37.2)
≥ 80 years	71 (22.7)	76 (25.9)
Female	169 (54.0)	163 (55.6)
Standard of care antibiotic		
Metronidazole	150 (47.9)	139 (47.4)
Vancomycin	152 (48.6)	148 (50.5)
Fidaxomicin	11 (3.5)	6 (2.0)
Clinical characteristics		
≥ 65 years of age^a^	184 (58.8)	185 (63.1)
Primary CDI	209 (66.8)	193 (65.9)
≥ 1 CDI episodes in past 6 months^a^	84 (26.8)	80 (27.3)
1 previous CDI episode ever	57 (18.2)	40 (13.7)
≥ 2 previous CDI episodes ever	40 (13.1)	51 (18.0)
Severe CDI (Zar score ≥ 2)^a,b^	57 (18.2)	57 (19.5)
Immunocompromised^a,c^	85 (27.2)	59 (20.1)
Inpatient at time of randomization	265 (84.7)	256 (87.4)
Antibiotic use^d^ during SOC	123 (39.3)	115 (39.2)
Antibiotic use^d^ after SOC	102 (32.6)	95 (32.4)
Charlson index ≥ 3	145 (46.3)	128 (43.7)
Renal impairment^e^	60 (19.2)	40 (13.7)
Hepatic impairment^f^	25 (8.0)	16 (5.5)
Albumin < 2.5 g/dL	57 (18.2)	48 (16.4)
*C. difficile* strain^g^		
Participants with a positive baseline culture	191	179
Ribotype 027, 078, or 244 strain^a^	37 (19.4)	45 (25.1)
Ribotype 027 strain	28 (14.7)	36 (20.1)
Prespecified risk factors		
0 risk factors	58 (18.5)	51 (17.4)
Participants with ≥ 1 risk factor	255 (81.5)	242 (82.6)
1 risk factor	107 (42.0)	116 (47.9)
2 risk factors	111 (43.5)	82 (33.9)
≥3 risk factors	37 (14.5)	44 (18.2)

^a^Prespecified risk factor

^b^Based on the following: (1) age > 60 years (1 point), (2) body temperature > 38.3 °C (> 100 °F) (1 point), (3) albumin level < 2.5 g/dL (1 point), (4) peripheral WBC count > 15,000 cells/mm^3^ within 48 h (1 point), (5) endoscopic evidence of pseudomembranous colitis (2 points), and (6) treatment in an intensive care unit (2 points)

^c^Defined on the basis of a subject’s medical history or use of immunosuppressive therapy

^d^Systemic antibiotic other than SOC antibiotic given to treat CDI

^e^Renal impairment defined as serum creatinine ≥ 1.5 mg/dL

^f^Hepatic impairment defined by two or more of the following: (1) albumin ≤ 3.1 g/dL, (2) ALT ≥ 2× ULN, (3) total bilirubin ≥ 1.3× ULN, or (4) mild, moderate, or severe liver disease (as reported on the Charlson index)

^g^Denominator is subjects in the mITT population with a positive baseline culture

**Fig. 2:**
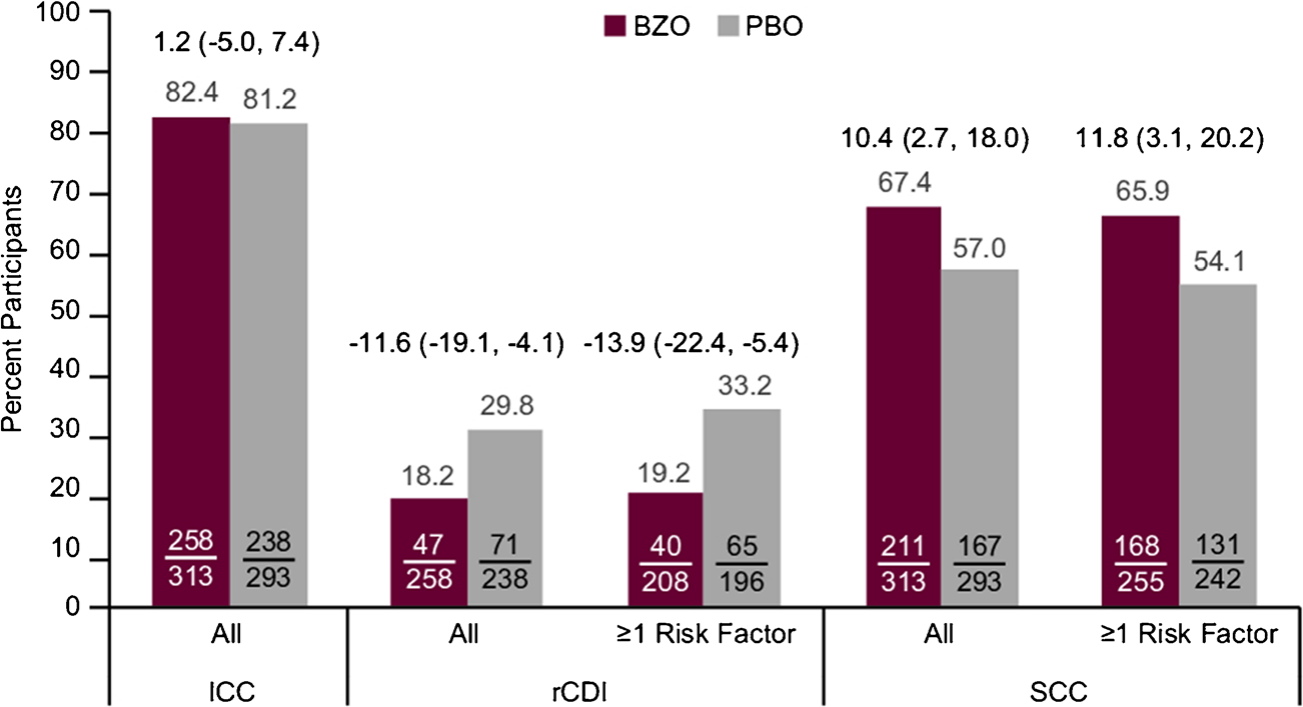
Proportion of participants with ICC and SCC (mITT population) and rCDI (clinical cure population). Numbers above bars indicate difference and 95% confidence interval. All, all European participants; ≥ 1 risk factor, European participants with at least 1 risk factor for CDI recurrence (≥ 65 years of age, ≥ 1 CDI episodes in past 6 months, severe CDI (Zar score ≥ 2), immunocompromised, ribotypes 027, 078, or 244 strain; BEZ, bezlotoxumab; CI, confidence interval; ICC, initial clinical cure; mITT, modified intent-to-treat; PBO, placebo; rCDI, recurrent *Clostridium difficile* infection; SCC, sustained clinical cure

The rate of ICC was similar between treatment groups (bezlotoxumab vs placebo 82.4% vs 81.2%; difference [95% CI] 1.2 [− 5.0, 7.4]; Fig. 2). The rate of rCDI was lower in the bezlotoxumab group compared with the placebo group overall (bezlotoxumab vs placebo 18.2% vs 29.8%; difference [95% CI] − 11.6 [− 19.1, − 4.1]), and among those with ≥ 1 risk factor for rCDI (bezlotoxumab vs placebo 19.2% vs 33.2%; difference [95% CI] − 13.9 [− 22.4, − 5.4]; Fig. 2). The rate of SCC was higher in the bezlotoxumab group compared with the placebo group overall (bezlotoxumab vs placebo 67.4% vs 57.0%; difference [95% CI] 10.4 [2.7, 18.0]), and among those with ≥ 1 risk factor for rCDI (bezlotoxumab vs placebo 65.9% vs 54.1%; difference [95% CI] 11.8 [3.1, 20.2]; Fig. 2).

**Fig. 3:**
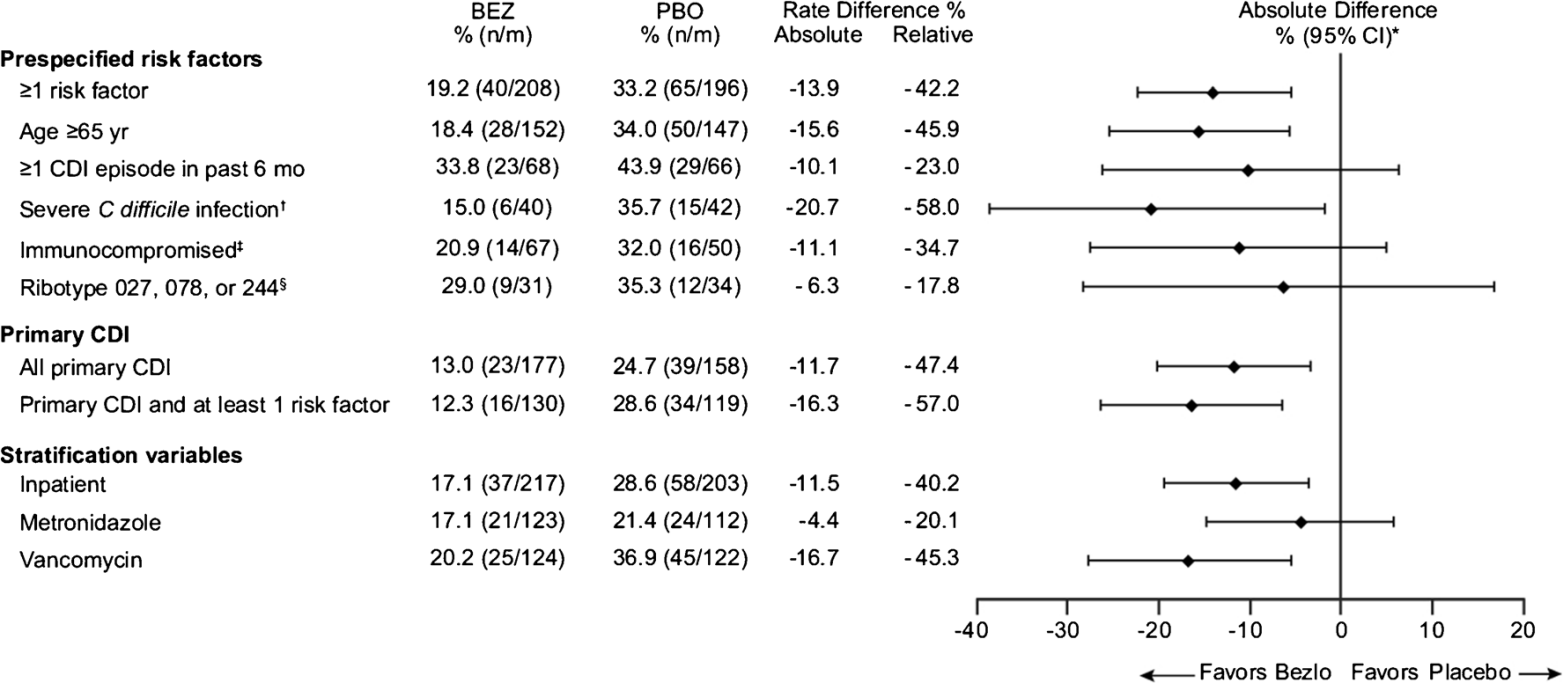
*C*. *difficile* infection recurrence rates by risk factor subgroup in European participants (clinical cure population). Unless otherwise specified, each subgroup includes all patients with the risk factor(s) (i.e., those with only the specified risk factor[s] and those with the specified risk factor[s] and ≥ 1 additional risk factor). CDI Hx, *Clostridium difficile* infection history in the previous 6 months; CI, confidence interval. ^a^Based on Miettinen and Nurminen method without stratification. ^b^Zar score ≥ 2 based on the following: (1) age > 60 years (1 point); (2) body temperature > 38.3 °C (> 100 °F) (1 point); (3) albumin level ˂ 2.5 mg/dL (1 point); (4) peripheral WBC count > 15,000 cells/mm3 within 48 h (1 point); (5) endoscopic evidence of pseudomembranous colitis (2 points); and (6) treatment in an intensive care unit (2 points). ^c^Defined on the basis of a subject’s medical history or use of immunosuppressive therapy. ^d^Denominator is subjects in the mITT population with a positive culture

Figure 3 shows the rCDI rates by risk factor subgroups. Compared with placebo, treatment with bezlotoxumab was associated with a lower rate of rCDI in participants with ≥ 1 prespecified risk factor for rCDI (33% vs 19%, respectively; difference − 14%) particularly in those ≥ 65 years of age (34% vs 18%, respectively; difference − 16%) and with severe CDI (36% vs 15%, respectively; difference − 21%). Compared with placebo, treatment with bezlotoxumab was associated with a lower rate of rCDI in all participants with primary CDI (25% vs 13%, respectively; difference − 12%), with a larger difference observed in participants with primary CDI who had at least 1 risk factor for rCDI (29% vs 12%, respectively; difference − 16%). Participants who were hospitalized at the time of infusion and those who were treated with vancomycin experienced a lower rate of rCDI when treated with bezlotoxumab compared with placebo (Fig. 3). In other subgroups, the difference favored bezlotoxumab; however, confidence intervals were wide and included zero, most likely due to the small sample sizes (Fig. 3).

**Fig. 4:**
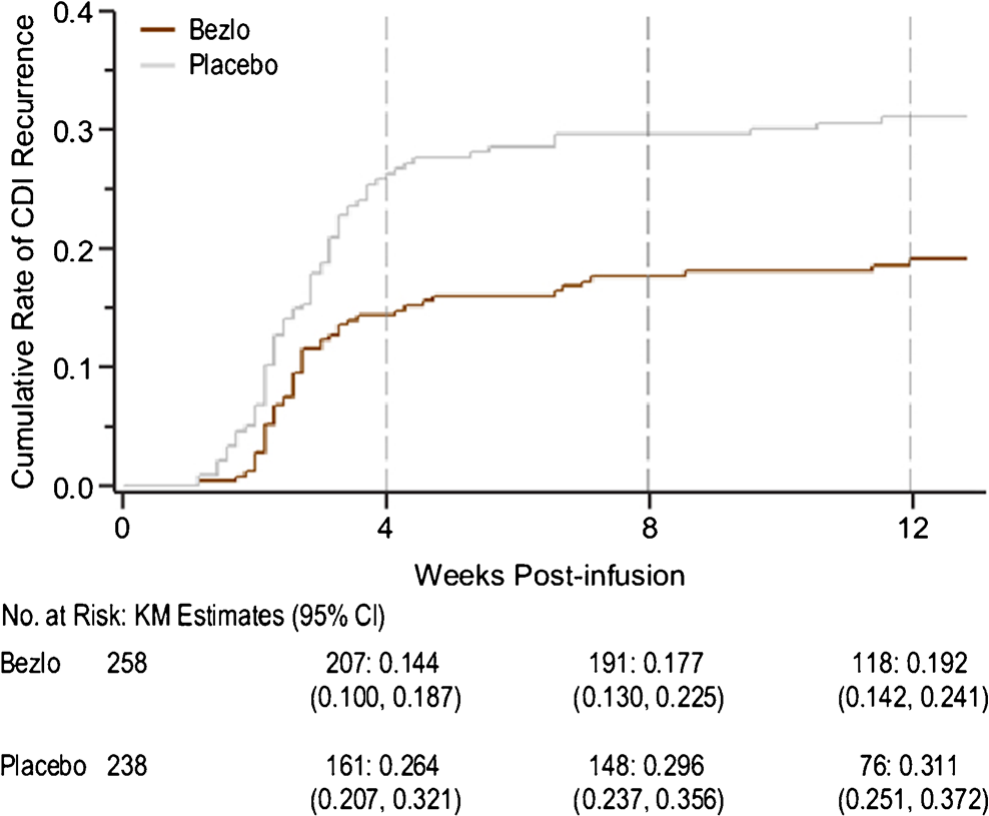
Kaplan-Meier plot of time to CDI recurrence

The week 12 Kaplan-Meier CDI recurrence event rate was lower in the bezlotoxumab treatment group compared with the event rate in the placebo group (Fig. 4). The majority (approximately 82%) of all recurrences occurred within the first 4 weeks following the infusion in both treatment groups (Fig. 4). The differences in the distributions of times to CDI recurrences between the bezlotoxumab group and the placebo group were observed as early as 3 weeks post-infusion, and this continued to increase throughout the 12-week follow-up period (Fig. 4).

In participants who experienced an episode of rCDI during the 12-week follow-up period, diarrhea duration was generally similar between groups. However, a higher proportion of placebo participants had severe disease (13% in the placebo group vs 9% in the bezlotoxumab group), and a higher percentage of placebo participants were treated with a CDI antibiotic for the recurrence compared with bezlotoxumab participants (70% vs 66%; Table 2). Bezlotoxumab participants had a lower proportion of CDI-associated rehospitalization within 30 days of discharge compared with placebo participants (5% vs 15%; Table 3), while all-cause rehospitalizations and the proportion of participants who died within 30 days or 90 days of randomization were similar between groups (Table 3).

**Table 2:** Time to resolution, severity, and treatment of rCDI episode (clinical cure population who experienced rCDI during the 12-week follow-up period)

	Bezlotoxumab (*n* (%))	Placebo (*n* (%))
	*n* = 47	*n* = 71
Diarrhea severity and duration		
Maximum number of loose stools during CDI episode (median)	5 (10.6)	6 (8.5)
Time to resolution of new episode (days)		
≤ 2	24 (51.1)	34 (47.9)
3 or more	23 (48.9)	37 (52.1)
Severe CDI (Zar score ≥ 2)	4 (8.5)	9 (12.7)
Any CDI antibiotic treatment^a^	31 (66.0)	50 (70.4)

^a^Any vancomycin, metronidazole, or fidaxomicin

**Table 3:** Additional outcomes in European participants

	Bezlotoxumab (*n* (%))	Placebo (*n* (%))
	*n* = 265	*n* = 256
30-day rehospitalization^a^		
All-cause	61 (23.0)	68 (26.6)
CDI-associated	13 (4.9)	38 (14.8)
Death^b^		
30-day mortality	16 (5.1)	15 (5.1)
90-day mortality	31 (9.9)	31 (10.5)

^a^mITT patients who were inpatients at randomization

^b^APaT, all patients as treated population

## Discussion

The results of this post hoc analysis used pooled data to evaluate the efficacy of bezlotoxumab treatment in European participants enrolled in the MODIFY I and II trials. The results of this analysis were consistent with the primary results from MODIFY I and II, which demonstrated that treatment with bezlotoxumab reduced CDI recurrence over a 12-week period compared with placebo in participants overall and in those with at least 1 risk factor [14].

*C. difficil*e has multiple strain types, some of which are associated with greater virulence, more severe disease, and increased complications and recurrences [21–23]. The EUCLID surveillance study identified 125 different ribotypes in 19 countries in Europe [18], while a study conducted by the Centers for Disease Control identified 143 distinct ribotypes in the USA [15]. The most commonly identified strains in both Europe and North America include ribotypes 001, 014, 020, 027, and 078; however, the prevalence differs between the two continents [15–18, 24]. The rates of ribotype 027 decreased in prevalence in the USA and Canada between 2009 and 2015, but ribotype 027 was more prevalent in Eastern European countries in 2015 compared with 2009 while the remaining unchanged in other parts of Europe [25]. Importantly, the differences in strain types and the differences in their distribution between North America and Europe did not appear to impact the efficacy of bezlotoxumab in reducing the rate of rCDI in the European population compared with the global population enrolled in the MODIFY trials.

Hospital costs account for the majority of costs in CDI and rCDI cases [26]. Therefore, the longer hospital stays and higher readmission rates associated with rCDI have a significant adverse economic impact in many European countries. In Germany, the mean length of hospital stay for a rCDI infection, often associated with admission to ICU, is 94 days at a cost of €73,900 (95% CI €50,340, €97,460) compared with 32 days for CDI at a cost of €18,460 (95% CI €14,660, €22,270) [5]. A second study conducted in 6 UK hospitals showed that the median cost of rCDI infection was £7539 (IQR £5617, £9730) for a mean length of stay of 21 days compared with £6294 (IQR £2700, £9216) for a length of stay of 15.5 days for a first CDI episode [26]. In the current analysis of European participants, there was a greater proportion of hospitalized patients compared with the overall population (86% vs 68%). In participants who were hospitalized at randomization, the rate of 30-day rehospitalizations associated with CDI was lower in the bezlotoxumab treatment group compared with the placebo group. This is consistent with a similar post hoc analysis of the MODIFY trials, which demonstrated that bezlotoxumab-treated inpatients experienced fewer CDI-associated readmissions during the 30 days after discharge compared with placebo-treated inpatients [27].

There were some limitations of this analysis: First, it was a post hoc analysis, so it was not powered to assess statistical significance. The results should be interpreted as hypothesis generating. Secondly, the possible effect of bezlotoxumab on the resolution of symptoms of the episode under treatment at the time of randomization could not be evaluated because the majority of participants had been receiving antibiotics for at least 3 days prior to receiving bezlotoxumab or placebo and symptoms had already resolved. In addition, not all regions in all countries across Europe were represented in the MODIFY trials; therefore, the results may not be generalizable to all patients living in the European region. However, the participants included in the analysis did represent the majority of the region and likely included the most common *C. difficile* strains found in the European region.

In conclusion, treatment with bezlotoxumab reduced CDI recurrence over a 12-week period and was associated with fewer CDI-associated rehospitalizations within 30 days of discharge in European participants, with the presumed reduction in the economic cost of patient management. Therefore, it is reasonable to consider the use of bezlotoxumab as an adjunct to standard of care antibacterial therapy in European patients with one or more risk factors for rCDI.
